# Interaction and Mutual Activation of Different Innate Immune Cells Is Necessary to Kill and Clear Hepatitis C Virus-Infected Cells

**DOI:** 10.3389/fimmu.2017.01238

**Published:** 2017-09-29

**Authors:** Volker Klöss, Oliver Grünvogel, Guido Wabnitz, Tatjana Eigenbrod, Stefanie Ehrhardt, Felix Lasitschka, Volker Lohmann, Alexander H. Dalpke

**Affiliations:** ^1^Department of Infectious Diseases, Medical Microbiology and Hygiene, University Hospital Heidelberg, Heidelberg, Germany; ^2^Department of Infectious Diseases, Molecular Virology, University of Heidelberg, Heidelberg, Germany; ^3^Section Molecular Immunology, Institute of Immunology, University of Heidelberg, Heidelberg, Germany; ^4^Institute of Pathology, University Hospital Heidelberg, Heidelberg, Germany

**Keywords:** hepatitis C virus, natural killer cell, monocyte, plasmacytoid dendritic cell, interferon, tumor necrosis factor-related apoptosis inducing ligand

## Abstract

Innate immune cells can sense hepatitis C virus (HCV)-infected cells and respond with anti-viral actions including secretion of interferons (IFNs). In previous studies, the response of individual innate immune cells against HCV was analyzed in detail. We hypothesized that interaction of multiple innate immune cells increases the magnitude of the immune response and eventually leads to clearance of HCV-infected cells. To investigate this, we co-cultured Huh-7 HCV subgenomic replicon (SGR) cells with peripheral blood mononuclear cells (PBMCs). We confirm secretion of IFNα by plasmacytoid dendritic cells (pDCs) and IFNγ by natural killer (NK) cells in the co-culture setup. Moreover, we observed that also monocytes contribute to the anti-viral response. Flow cytometry and ImageStream analysis demonstrated that monocytes take up material from HCV SGR cells in co-culture with PBMCs. Preceding the uptake, PBMCs caused apoptosis of HCV SGR cells by tumor necrosis factor-related apoptosis inducing ligand (TRAIL) expression on NK cells. We observed that only the interplay of monocytes, pDCs, and NK cells resulted in efficient clearance of HCV SGR cells, while these cell populations alone did not kill HCV SGR cells. Despite similar TRAIL receptor expression on Huh-7 control cells and HCV SGR cells, HCV activated PBMCs specifically killed HCV SGR cells and did not target Huh-7 control cells. Finally, we showed that HCV replicating cells *per se* are sensitive toward TRAIL-induced apoptosis. Our results highlight the importance of the interplay of different innate immune cells to initiate an efficient, rapid, and specific response against HCV-infected cells.

## Introduction

Hepatitis C virus (HCV) is a positive-strand RNA virus belonging to the family of Flaviviridae. Worldwide about 130–170 million people are infected by HCV and while around 25% of patients with acute infection spontaneously clear HCV, 75% develop chronic infection with risk for severe liver disease (1). The innate immune system, as the first line of defense, plays an important role for the immediate response against the invading pathogen. Indeed, several innate immune cells are present in or migrate to the liver upon HCV infection. Among others, plasmacytoid dendritic cells (pDCs) are enriched in HCV-infected livers (2). While in peripheral blood mononuclear cells (PBMCs) around 12% of the lymphocytes are natural killer (NK) cells, in the liver, this number rises to 30% (3). Moreover, 15–25% of the cells in the liver are Kupffer cells, the resident liver macrophages (4, 5).

In recent years, activation of innate immune cells by HCV has drawn attention. As a model system Takahashi et al. co-cultured HCV-infected cells with PBMCs and observed secretion of IFNα by pDCs (6). They recognized that this rapid activation could also be induced with HCV subgenomic replicon cells (HCV SGR) and that pDCs sense viral HCV RNA *via* TLR7. Later, it was shown that also monocytes and NK cells respond to HCV-replicating cells (7). Noteworthy, IFNγ production by NK cells is dependent on monocytes (7) and on pDCs (8). Secretion of interferons (IFNs) in this co-culture is an important anti-viral mechanism, as IFNs stimulate the induction of interferon-stimulated genes, thereby inhibiting further viral replication (9–11). So far, these studies showed that multiple innate immune cells are activated by HCV and can limit viral replication. However, studies were limited to the analysis of the response of individual immune cell populations against HCV. Hence, most of the experiments were conducted with purified immune cells, yet interactions between innate immune cells will take place and probably are important for the overall activation state, as shown for NK cell activation by monocytes and pDCs (7, 8). We speculated that multiple interactions between different innate immune cells augment the overall activation state and thus exert a stronger anti-viral response.

In this study, we used co-culture systems of liver cell lines with acute and persistent HCV replication and PBMCs to investigate whether the interaction of multiple innate immune cells results in an efficient anti-viral response. While IFNs can limit HCV replication, we hypothesized that mutual interaction and activation between innate immune cells can lead to killing and clearance of HCV SGR cells. Since innate immune cells in the context of HCV infection are suspected to cause liver injury (12), we analyzed if HCV activated innate immune cells show specificity for targeting only HCV-infected cells.

## Materials and Methods

### Reagents, Inhibitors, and Blocking Antibodies

R848 was purchased from InvivoGen (San Diego, CA, USA), lipopolysaccharide (LPS) from Salmonella minnesota was kindly provided by U. Seydel (Division of Biophysics, Research Center Borstel, Borstel, Germany). Bafilomycin was from Calbiochem (Darmstadt, Germany). The pan-Caspase inhibitor Z-VAD-FMK was from InvivoGen, Caspase-8 inhibitor Z-IETD-FMK and Caspase-1 inhibitor Z-YVAD-FMK from Enzo Life Sciences (Lausen, Switzerland). TRAIL blocking antibody was from BD (Heidelberg, Germany, 550912) as well as the appropriate IgG control antibody (BD, 553447).

### Cells

All Huh-7- and Huh-6-derived cell lines were cultured in Dulbecco’s modified Eagle medium (DMEM) supplemented with 10% fetal bovine serum, 100 U/ml of penicillin, 100 ng/ml of streptomycin and non-essential amino acids (all from Thermo Fisher Scientific, Waltham, MA, USA). Cells were cultivated at 37°C and 5% CO_2_. Naïve Huh-7 and Huh-7 9–13 cells harboring the HCV genotype 1b replicon Con1 were described previously (13) (GenBank accession no. AJ238799), the latter are designated Huh-7 Con1 throughout this study. Cured Con1 cells were generated by IFNα treatment of Huh-7 Con1 cells as described (14). Naïve Huh-6, Huh-6 JFH (HCV genotype 2a, AB047639) have been described (15), the latter were cured by treatment with direct acting antivirals (unpublished, A. Cerwenka, DKFZ, Heidelberg, Germany). Huh-7 cells with SGRs from dengue virus (Huh-7 DV, KU725663) (16) or from hepatitis A virus (Huh-7 HAV, M59808) (17) have been described before. Huh-7.5 cells were a kind gift by C. Rice (The Rockefeller University, New York, NY, USA) (18).

### PBMC Isolation

Fresh human PBMCs were isolated from blood from voluntary healthy donors by standard Pancoll density-gradient centrifugation (PAN-Biotech GmbH, Aidenbach, Germany). PBMCs were directly cultured in RPMI 1640 (Biochrom, Berlin, Germany) supplemented with 10% fetal bovine serum. Overall blood from 30 different donors was used, yet individual experiments were done with 3–5 donors as indicated in the respective figure legend. Donors had no history of hepatitis. Blood sampling was approved by the ethics committee of the Medical Faculty Heidelberg and was done in accordance with their recommendations. All subjects gave written informed consent in accordance with the Declaration of Helsinki.

### Selection of SGR Cells

To ensure constant selection for replicon cells, Huh-7 Con1 cells were cultured with 1 mg/ml of G418 (Carl Roth, Karlsruhe, Germany), Huh-6 JFH cells with 0.5 mg/ml of G418, Huh-7 DV cells with 75 µg/ml Hygromycin B (Thermo Fisher Scientific), and Huh-7 HAV cells with 2.5 µg/ml of Blasticidin S (Thermo Fisher Scientific).

### Co-Culture Setup

For the co-culture of hepatoma cells with PBMCs, 1 × 10^5^ Huh cells were seeded in 48-well plates in DMEM. Upon adherence of Huh cells (after 4 h), DMEM was removed and 1 × 10^6^ PBMCs were added in RPMI. After overnight co-culture supernatant or cells were harvested for further analysis. When purified subsets of specific immune cells were used for co-culture with Huh cells, 1 × 10^4^ pDCs, 1 × 10^5^ NK cells, or 1 × 10^5^ monocytes were used. Accordingly, when these cells were depleted from PBMCs, those numbers were subtracted from 1 × 10^6^ PBMCs. In triple co-culture experiments, 5 × 10^4^ Huh-7 cells were seeded together with 5 × 10^4^ Huh-7 Con1 cells.

For flow cytometry analysis of the uptake of hepatoma cell remnants by monocytes, Huh-7 cells were stained with carboxyfluorescein succinimidyl ester (CFSE) (Sigma-Aldrich, Taufkirchen, Germany) or with Cytotell^TM^ RED (CytoRED) (AAT Bioquest, Sunnyvale, CA, USA) prior to seeding. Cells were incubated with the intracellular dye CFSE (4.5 µM in PBS) for 5 min at room temperature in the dark, washed once with PBS, once with DMEM and then were seeded. Using the same protocol hepatoma cells were also stained with Cytotell^TM^ RED (CytoRED) (AAT Bioquest, Sunnyvale, CA, USA) with a 0.5× dye working solution for triple co-culture experiments.

### Purification of Immune Cell Subsets

Plasmacytoid dendritic cells, NK cells, and monocytes were purified or depleted from PBMCs by MACS separation technology with CD304, CD56, or CD14 specific magnetic beads, respectively (Miltenyi Biotec, Bergisch Gladbach, Germany).

### Cell Stimulation and HCV Infection

Purified monocytes were co-cultured with Huh-7 cells in the presence of 700 pg/ml IFNα (Peprotech, Hamburg, Germany) or 7 ng/ml IFNγ (Peprotech). To analyze sensitivity of Huh-7 cells for TRAIL and TNFα stimulation, Huh-7 cells were treated with 50 or 100 ng/ml of TRAIL (eBioscience) or TNFα (Peprotech) for the indicated time points (see figures).

For infection of Huh-7 cells with HCV, the JC1 virus was used as described (19). 5 × 10^4^ Huh-7 cells were seeded in a 24-well plate. 24 h later infection with a multiplicity of infection of 3 was performed for 6 h. Cells were then washed and stained with CFSE as described above. After 72 h, PBMCs were added to infected Huh-7 cells for overnight co-culture.

### Cytokine Secretion and Cytotoxicity

Cytokine secretion was quantified by Sandwich-ELISA. Cell-free supernatants were harvested and IFNα, IFNγ (both eBioscience, Frankfurt, Germany), and interleukin (IL)-6 (BD, Heidelberg, Germany) levels were measured. Cell-free supernatants were also used to measure immune cell mediated cytotoxicity of Huh-7 cells by detection of lactate dehydrogenase (LDH) release with the Cytotoxicity Detection Kit^Plus^ (Roche, Mannheim, Germany).

### Flow Cytometry Antibodies

Analysis of CD80 or CD86 expression on monocytes was done with CD14-APC (BD, 555399), CD80-PE (BD, 557227), or CD86-PE (BD, 555665). For detection of CD25, CD69, and TRAIL on NK cells, NK cells were identified as CD56^+^/CD3^−^. The following stainings were performed: CD25-PerCP-Cy5.5 (Biolegend, San Diego, CA, USA, 356112), CD56-APC (Miltenyi Biotec, 130-090-843), and CD3-FITC (BD, 555332). CD69-APC (BD, 555533), CD56-Pe-Cy7 (eBioscience, 25-0567-42), and CD3-FITC (BD, 555332). TRAIL-PE (BD, 550516), CD56-APC (Miltenyi Biotec, 130-090-843), and CD3-APC-Cy7 (BD, 557832). Samples were measured on a BD FACSCanto II with BD FACSDIVA 8 software.

### Classical and Imaging Flow Cytometry Analysis

To analyze the uptake of CFSE positive Huh cells by monocytes, monocytes were stained with CD14-PE (BD, Heidelberg, Germany, 562691) in FACS buffer (2% FCS in PBS) and uptake was quantified by classical flow cytometry. Samples were measured on a BD FACSCanto II with BD FACSDIVA 8 software.

Imaging flow cytometry (hereafter termed ImageStream) was performed as described (20). Cells were analyzed by ImageStream (Amnis, Merck Millipore, Darmstadt, Germany). Further antibodies used for flow cytometry are described in Data Sheet S1 in Supplementary Material.

### Western Blot Analysis

To analyze induction of apoptosis in Huh-7 cells cleaved poly (ADP-ribose) polymerase (PARP) was detected by Western blot. After co-culture with PBMCs or incubation with recombinant TRAIL, Huh-7 cells were washed once with PBS, lysed in Laemmli buffer, and incubated at 95°C for 10 min. Proteins were separated by SDS-PAGE and transferred to a nitrocellulose membrane by semidry blotting. Unspecific binding was blocked by incubating the membranes in 5% dry milk in 1xTBST (TBS, 0.05% Tween-20) for 1 h. Primary antibodies against cleaved PARP (Cell Signaling, Leiden, Netherlands, #9541) and β-actin (Cell Signaling, #4970) were diluted in blocking buffer 1:1,000 and 1:5,000, respectively. Membranes were incubated with primary antibody overnight at 4°C, washed three times for 10 min with 1× TBST at RT, and incubated with HRP-conjugated anti rabbit antibody (Cell Signaling, #7074) diluted 1:4,000 in blocking buffer for 45 min at RT. Again, membranes were washed as described above and proteins were then detected by using enhanced chemiluminescence (ECL) substrate (PerkinElmer, Rodgau, Germany). Densitometric analysis was performed using ImageJ software (National Institutes of Health, Bethesda, MD, USA). Original image data are shown as attachment in the Data Sheet S1 in Supplementary Material.

### Quantitative RT-PCR

Cellular RNA was extracted with the peqGold Total RNA Kit (peqlab Biotechnology, Erlangen, Germany). RNA was transcribed into cDNA using High Capacity cDNA RT Kit (Applied Biosystems, Foster City, CA, USA). RT-PCR was set up with SYBR^®^Green PCR Master Mix Fast (Applied Biosystems) and specific primers for each target (DR4 forward: 5′ AGAGAGAAGTCCCTGCACCA 3′, DR4 reverse: 5′ GTCACTCCAGGGCGTACAAT 3′, DR5 forward: 5′ AAGACCCTTGTGCTCGTTGT 3′, DR5 reverse: 5′ AGGTGGACACAATCCCTCTG 3′, DcR2 forward: 5′ TACCACGACCAGAGACACC 3′, DcR2 reverse: 5′ CACCCTGTTCTACACGTCCG 3′, β-actin forward: 5′ GGCTCCGATATCTCTGTCGT 3′, β-actin reverse: 5′ ATGTTGCATTTCGTCACACC 3′). Analysis was performed on a StepOne Plus RT-PCR cycler (Applied Biosystems). Specificity of PCR was controlled by no-template and no-RT control samples and by melting curves. The relative expression of each target gene as compared to β-actin was calculated.

### Statistics

Experiments were repeated as indicated in the figure legends. Data are shown as mean + SD. Statistical significance was analyzed by ANOVA using R 3.0.2 (R Foundation for Statistical Computing, Vienna, Austria) followed by a two-sided Tukey *post hoc* test. Significant differences were considered at p values of less than 0.05 (*), 0.01 (**), 0.001 (***), no significance is indicated by ns.

## Results

### Plasmacytoid DCs and NK Cells Secrete IFNs in Response to HCV SGR Cells

**Figure 1:**
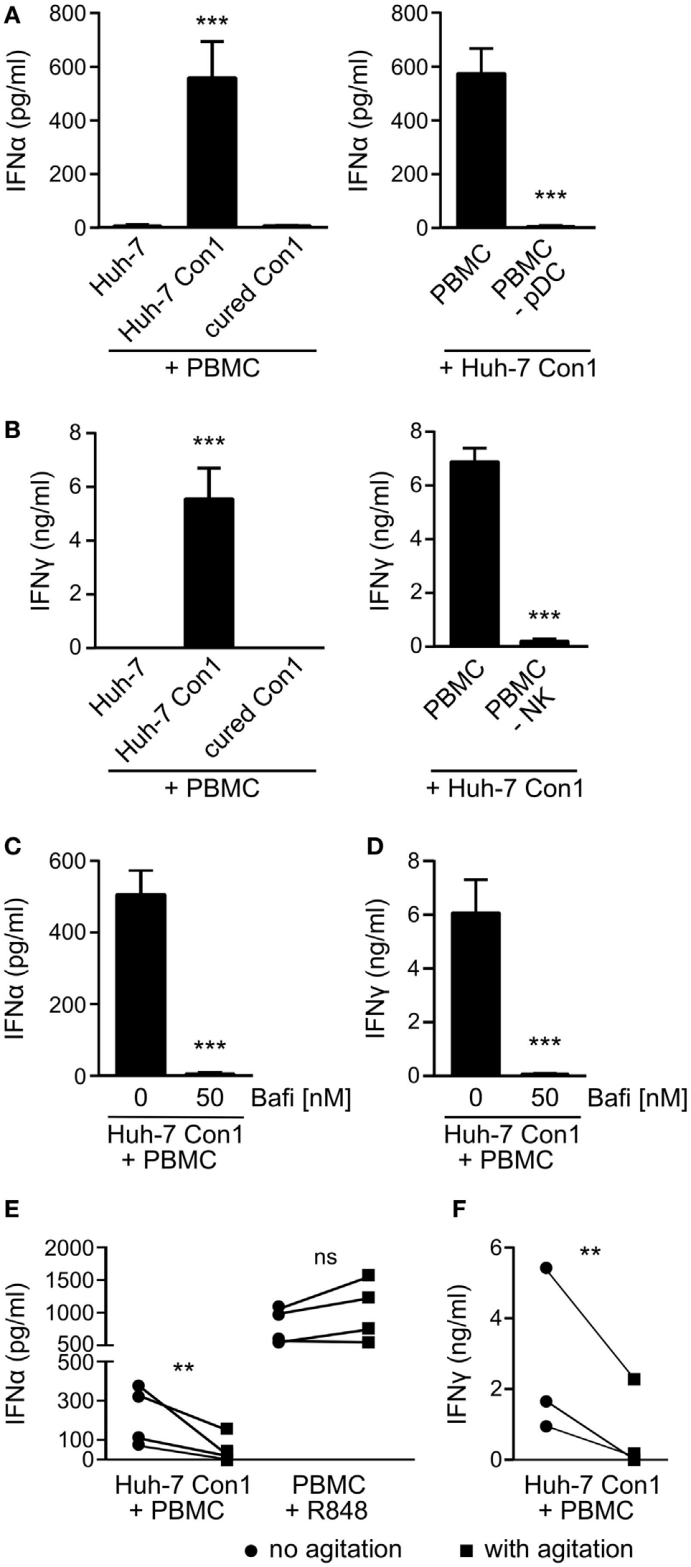
Plasmacytoid dendritic cells (pDCs) and natural killer (NK) cells are activated by hepatitis C virus subgenomic replicon cells. Huh-7 cells were co-cultured with peripheral blood mononuclear cells (PBMCs) overnight and cytokines measured by ELISA. **(A)** IFNα levels in co-cultures with PBMCs or with PBMCs depleted from pDCs (PBMC -pDC) (*n* = 3). **(B)** IFNγ levels in co-cultures with PBMCs or with PBMC depleted from NK cells (PBMC -NK) (*n* = 3). **(C,D)** IFNα and IFNγ secretion in PBMCs that were treated with Bafilomycin (Bafi) for 1 h before setting up co-cultures (*n* = 3). **(E,F)** IFNα and IFNγ levels in co-cultures that underwent continuous mechanical agitation (orbital shaker, 200 rpm) compared to co-cultures without agitation. PBMCs stimulated with R848 (1 µg/ml) were used as control.

Hepatitis C virus SGR cells (HCV SGR) have been reported to stimulate PBMCs in a process involving transfer of viral RNA from HCV SGR cells into PBMCs (6). To study the activation in more detail, we set up a co-culture system of HCV SGR cells and complete human PBMCs. In order to avoid potential transmission of viral RNA by viral particles, SGR cells were preferred over HCV-infected cells. We observed secretion of IFNα in co-cultures of the HCV SGR cell line Huh-7 Con1 with PBMCs (Figure 1A, left). IFNα was neither detected in co-cultures of Huh-7 control cells with PBMCs, nor when Huh-7 Con1 cells that were cured from the replicon (“cured Con1”) were used. Similar to the production of IFNα, IFNγ was only secreted in co-cultures of Huh-7 Con1 cells and PBMCs (Figure 1B, left).

To identify the cellular source of IFNα and IFNγ, distinct cell populations were depleted from PBMCs before stimulating with Huh-7 Con1 cells. Upon depletion of pDCs, IFNα was not secreted anymore in co-culture supernatants (Figure 1A, right). IFNγ production was abolished when NK cells were depleted from PBMCs (Figure 1B, right). The results show that pDCs and NK cells are activated by HCV SGR cells and respond by secreting IFNα and IFNγ, respectively.

We then investigated the mode of activation of PBMCs by HCV SGR cells. Earlier reports (6, 21) suggested a role for TLR7 recognizing viral RNA. Indeed, blocking nucleic acid recognizing endosomal TLRs using Bafilomycin resulted in complete loss of IFNα and IFNγ production (Figures 1C,D). Bafilomycin did not reduce IL-6 secretion by PBMCs treated with LPS (Figure S1 in Supplementary Material), triggering non-endosomal TLR4. Mechanistically, it was suggested that cell–cell contacts but also exosomes are required to initiate the innate immune response (6, 22). We observed no trans-activation when Huh-7 Con1 cells were separated from PBMCs by a Transwell insert nor could we stimulate PBMCs with concentrated supernatants of replicon cells (data not shown). Moreover, the production of IFNα and IFNy was significantly reduced when tight cell–cell contacts were inhibited by continuous mechanical shaking (Figures 1E,F). These findings support the hypothesis that innate immune cells are activated by viral RNA derived from infected hepatocytes in a manner that depends on close cell–cell contacts.

### Monocytes Take Up Material from HCV SGR Cells

**Figure 2:**
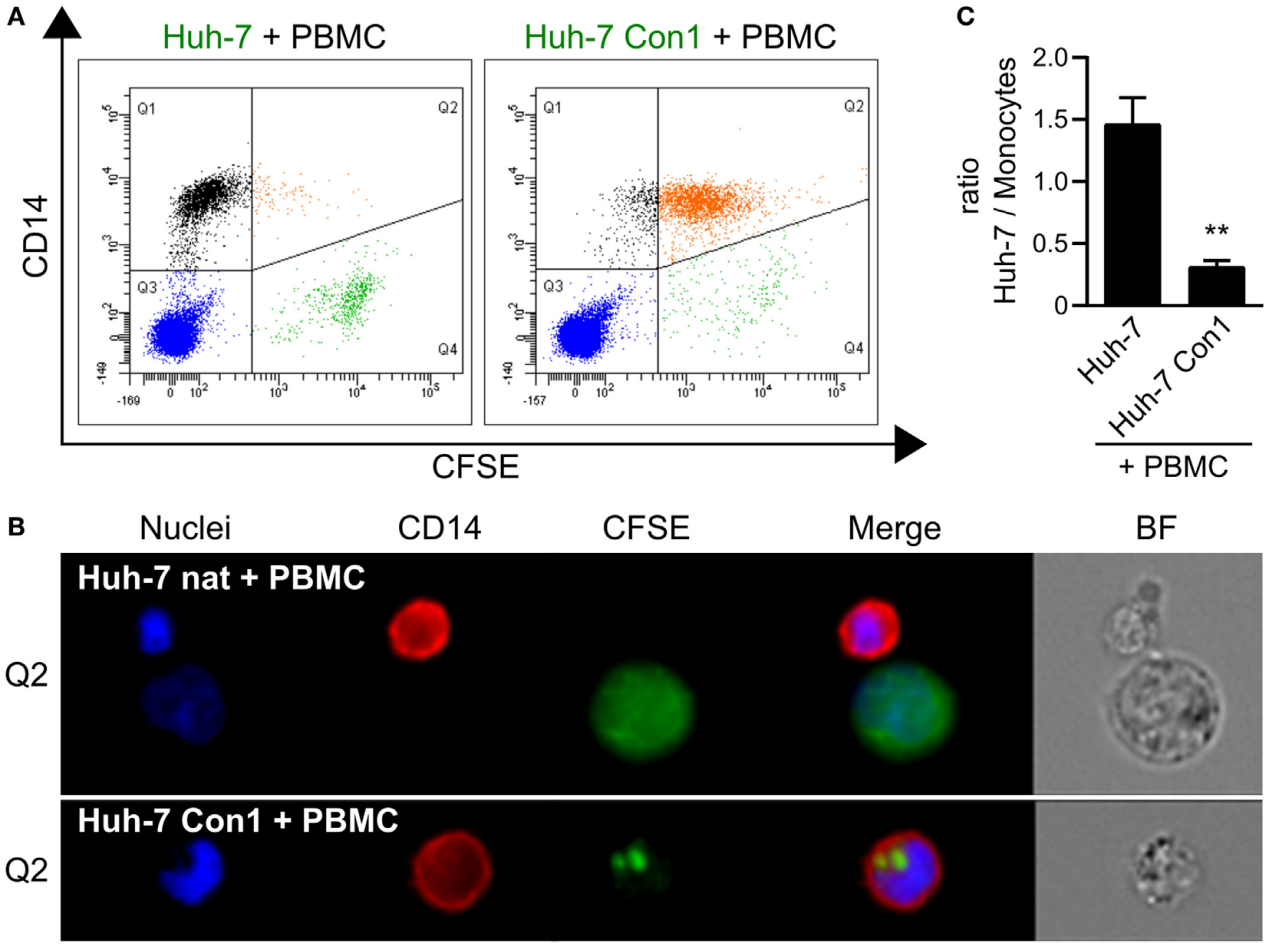
Phagocytosis of particles from hepatitis C virus subgenomic replicon cells by monocytes. Carboxyfluorescein succinimidyl ester (CFSE) stained Huh-7 cells were co-cultured with peripheral blood mononuclear cells (PBMCs). **(A)** Analysis of CFSE distribution in relation to monocytes (CD14^+^). **(B)** Uptake of Huh-7 cells by monocytes depicted by ImageStream analysis. **(C)** Ratio between Huh-7 cells and monocytes after co-culture based on flow cytometry analysis in **(A)** (*n* = 3).

During the characterization of cytokine responses of PBMCs with HCV SGR cells, we consistently observed by microscopy that the number of Huh-7 Con1 cells declined during the co-culture period (data not shown). Further investigations of this decline revealed a novel contribution of monocytes to the immune response against HCV SGR cells. When Huh-7 Con1 cells were stained with CFSE prior to addition of PBMCs, monocytes (identified by expression of CD14) became CFSE positive after overnight co-culture (Figure 2A, right plot). In contrast, monocytes were CFSE negative when PBMCs were co-cultured with CFSE labeled Huh-7 cells that lack the SGR (Figure 2A, left plot). To analyze this in detail, we performed ImageStream analysis. Co-culture of CFSE stained Huh-7 cells and PBMCs resulted in few double-positive cells (Figure 2A), which could be identified mostly as cell pairs (Figure 2B, upper row). In contrast, in the co-culture of Huh-7 Con1 cells with PBMCs, we observed that most of the cells double-positive for CD14 and CFSE consisted of monocytes that had taken up particles from Huh-7 Con1 cells (Figure 2B, lower row; Figure S2 in Supplementary Material). The size of these particles ranged from 1.8–3.3 µm in diameter, suggesting that activated PBMCs induced apoptosis of HCV SGR cells. Induction of apoptosis was supported by the observation that the number of Huh-7 Con1 cells significantly dropped compared to the number of Huh-7 cells in co-culture with PBMCs (shown in Figure 2A Q4, quantified in Figure 2C).

**Figure 3:**
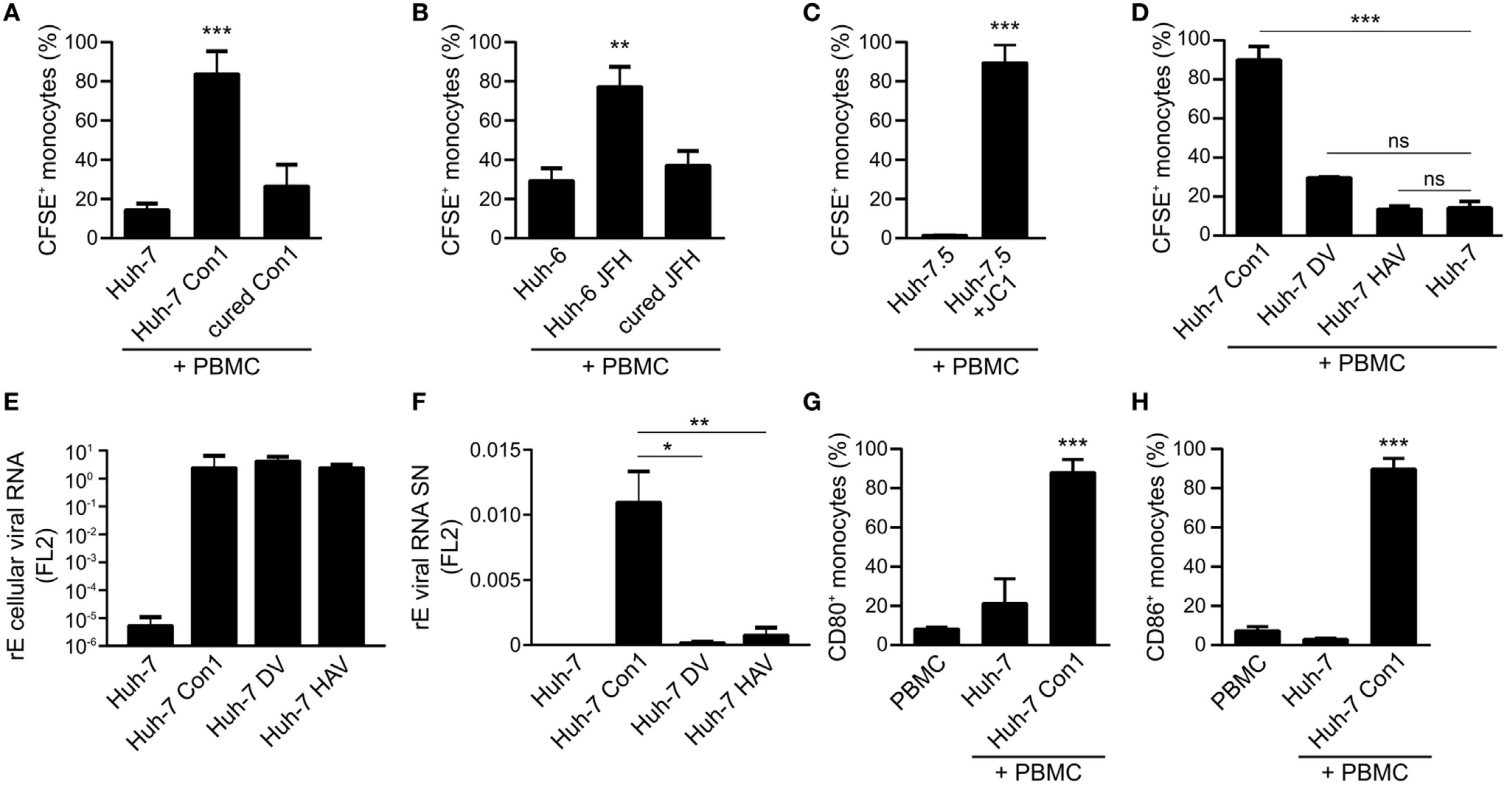
Phagocytosis of hepatitis C virus subgenomic replicon particles by monocytes is specific for HCV. **(A–D)** Peripheral blood mononuclear cells (PBMCs) were co-cultured with various carboxyfluorescein succinimidyl ester (CFSE)-stained Huh cells and the percentage of CFSE^+^ monocytes was determined by flow cytometry. **(A)** Co-cultures of PBMCs with Huh-7 cells (Con1 = HCV genotype 1b) (*n* = 3). **(B)** Co-cultures of PBMCs with Huh-6 cells (JFH = HCV genotype 2a) (*n* = 3). **(C)** Co-cultures of PBMCs with Huh-7.5 cells or with HCV-infected Huh-7.5 cells (Huh-7.5 + JC1) (*n* = 2). **(D)** Co-cultures of PBMCs with Huh-7 cells, Huh-7 Con1 (HCV), Huh-7 DV (dengue virus), or Huh-7 hepatitis A virus (HAV) SGR cells (*n* = 3). **(E,F)** Huh-7 and Huh-7 SGR cells (Con1, DV, and HAV) were seeded for quantification of intracellular viral RNA **(E)** and secreted viral RNA in the supernatant **(F)** was measured by qPCR. RNA lysis buffer was spiked with Firefly Luciferase 2 (FL2) *in vitro* transcript for normalization (*n* = 3). **(G,H)** Huh-7 cells were co-cultured with PBMCs overnight and monocytes were analyzed for activation by expression of CD80 and CD86 by flow cytometry (*n* = 4).

In order to quantify the uptake of CFSE stained Huh-7 cells by monocytes, the percentage of CFSE positive monocytes as well as the CFSE mean fluorescence intensity (MFI) of monocytes were analyzed. In co-cultures of Huh-7 Con1 cells with PBMCs 83% of the monocytes became CFSE positive, whereas in control cells this percentage was 14% with Huh-7 and 26% with cured Con1 cells (Figure 3A). To confirm these findings, another cell line containing a replicon of another HCV genotype (Huh-6 JFH, genotype 2a) was used with similar results (Figure 3B). As a proof-of-principle uptake experiments were also performed with HCV-infected cells instead of HCV SGR cells. Strikingly, the same phenotype was observed with HCV JC1-infected Huh-7.5 cells (Figure 3C). We next analyzed whether this uptake could also be observed with other RNA viruses. However, for dengue virus and HAV SGR cells, no significant uptake was observed (Figure 3D). Similar interpretations could be drawn when analyzing the MFI data (Figure S3 in Supplementary Material). We speculated that DV or HAV SGR cells secrete less viral RNA, thus explaining the lack of immune cell activation. Intracellular viral RNA levels were comparable in HCV, DV, and HAV SGR cells (Figure 3E). However, in contrast to HCV SGR cells, secretion of viral RNA by DV and HAV SGR cells indeed was significantly lower (Figure 3F). Of note, the uptake by monocytes was only observed with HCV SGR cells. Furthermore, we observed increased expression of the monocyte activation markers CD80 and CD86 in co-cultivation of Huh-7 Con1 cells with PBMCs (Figures 3G,H). Our data show that next to pDCs and NK cells also monocytes are activated by HCV SGR cells and contribute to the anti-viral response.

### Uptake of HCV SGR Cells Requires Presence of pDCs and NK Cells

**Figure 4:**
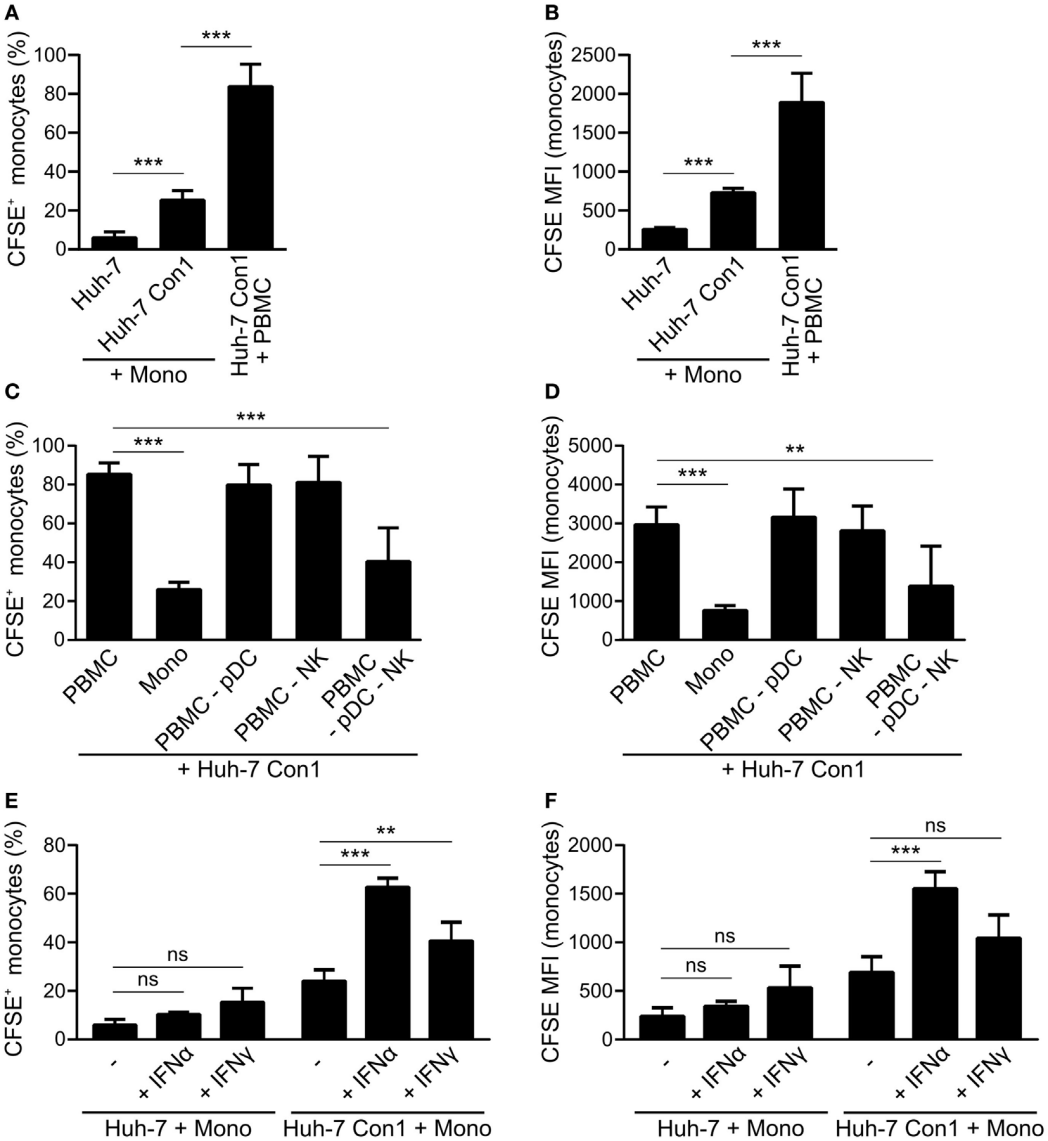
Plasmacytoid dendritic cells (pDCs) and natural killer (NK) cells promote phagocytosis of hepatitis C virus subgenomic replicon by monocytes. **(A,B)** Purified monocytes (Mono) or peripheral blood mononuclear cells (PBMCs) were co-cultured with carboxyfluorescein succinimidyl ester (CFSE) stained Huh-7 cells overnight. Percentage of CFSE^+^ monocytes **(A)** and CFSE mean fluorescence intensity (MFI) of monocytes **(B)** was determined (*n* = 5). **(C,D)** CFSE stained Huh-7 Con1 cells were co-cultured overnight with PBMCs, purified monocytes (Mono), PBMCs depleted from pDCs (PBMC -pDC), PBMCs depleted from NK cells (PBMC -NK) or with PBMC depleted from pDCs and NK cells (PBMC -pDC -NK). CFSE^+^ monocytes were determined (*n* = 3). **(E,F)** Purified monocytes were co-cultured with CFSE stained Huh-7 cells in presence of recombinant IFNα (700 pg/ml) or recombinant IFNγ (7 ng/ml). CFSE^+^ monocytes were determined (*n* = 4).

As monocytes within complete PBMCs were taking up Huh-7 Con1 cells, we addressed the question whether this phenotype was induced in a monocyte-intrinsic manner or whether it required additional cells. In co-cultures of purified monocytes with Huh-7 Con1 cells, 25% of monocytes became CFSE positive (6% with Huh-7, Figure 4A) and showed slightly increased CFSE MFI (Figure 4B). However, compared to monocytes within full PBMCs, the efficacy of uptake was clearly reduced with purified monocytes. Hence, we speculated that other innate immune cells contribute. As pDCs and NK cells were activated and secreted their respective IFNs in response to HCV SGR cells, we depleted those cells from PBMCs and analyzed the uptake of HCV SGR cell remnants by monocytes. Surprisingly, pDC or NK depletion alone did not reduce uptake. Only when both, pDCs and NK cells, were depleted the uptake by monocytes was significantly decreased (Figures 4C,D). Addition of recombinant IFNα or IFNγ (to mimic functional activities of either pDCs or NK cells) to the co-culture of purified monocytes with Huh-7 Con1 cells significantly increased the monocyte uptake (Figures 4E,F). Yet no synergistic effect was observed when combining both cytokines (data not shown). Neither IFNα nor IFNγ induced an uptake of Huh-7 control cells by monocytes, indicating that IFNs did not unspecifically kill Huh-7 cells. We show that monocytes by themselves respond to HCV SGR cells, but the uptake by monocytes is dramatically stronger in presence of pDCs and NK cells and can be mimicked by addition of IFNs.

### PBMCs Kill HCV SGR Cells

**Figure 5:**
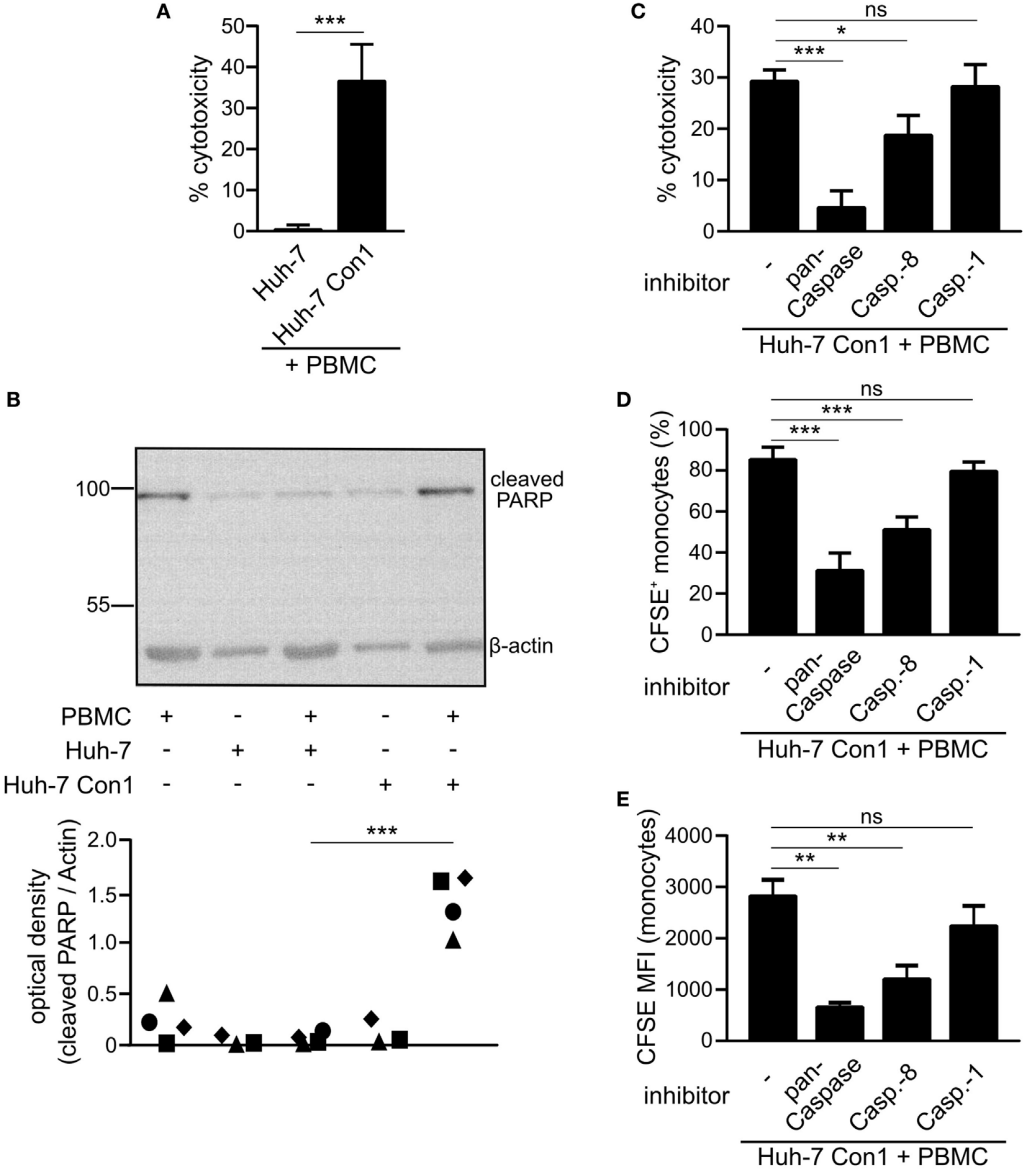
Killing of hepatitis C virus subgenomic replicon cells by peripheral blood mononuclear cells (PBMCs). Huh-7 cells were co-cultured with PBMCs overnight. **(A)** Supernatants were collected and measured for lactate dehydrogenase (LDH) activity (*n* = 5). **(B)** Cells were harvested and poly (ADP-ribose) polymerase (PARP) cleavage was analyzed by Western Blot (*n* = 3–4). **(C–E)** Huh-7 Con1 cells were co-cultured with PBMCs in presence of pan-Caspase inhibitor Z-VAD-FMK (20 µM), Caspase-8 inhibitor Z-IETD-FMK (20 µM), or Caspase-1 inhibitor Z-YVAD-FMK (20 µM). After overnight incubation LDH release was measured in the supernatant **(C)**, percentage of CFSE^+^ monocytes **(D)** and carboxyfluorescein succinimidyl ester (CFSE) mean fluorescence intensity (MFI) of monocytes **(E)** was determined by flow cytometry (*n* = 4–5).

ImageStream analysis showed that monocytes take up small particles of Huh-7 Con1 cells (Figure 2B). We speculated that those particles were remnants of Huh-7 Con1 cells that were killed by PBMCs. To support this hypothesis, an LDH release assay was performed. 36% of Huh-7 Con1 cells were killed in co-culture with PBMCs, while no LDH was released upon co-culture of PBMCs with Huh-7 control cells (Figure 5A). To analyze if Huh-7 Con1 cells undergo apoptosis in co-culture with PBMCs, PARP cleavage determining the final irreversible step of apoptosis was analyzed. Cleaved PARP was only detected in co-culture of PBMCs with Huh-7 Con1, but not with Huh-7 cells (Figure 5B). Since apoptosis in target cells is mediated by successive caspase activation, we tested if caspase inhibition protects Huh-7 Con1 cells from apoptosis. Inhibition of all caspases (pan-Caspase inhibitor) decreased killing of Huh-7 Con1 cells from 29 to 4.6% (Figure 5C). Blocking Caspase-8, which is the first caspase activated upon induction of apoptosis, reduced killing to 18%. Caspase-1, which is not involved in apoptosis but mediates inflammasome activation, had no effect on PBMC mediated killing of Huh-7 Con1 cells. In consequence, blocking of apoptosis reduced the uptake of CFSE labeled Huh-7 SGR cells by monocytes (Figures 5D,E), proving that killing of Huh-7 Con1 cells precedes the uptake by monocytes.

### Innate Immune Cells Interact to Mediate Killing of HCV SGR Cells

**Figure 6:**
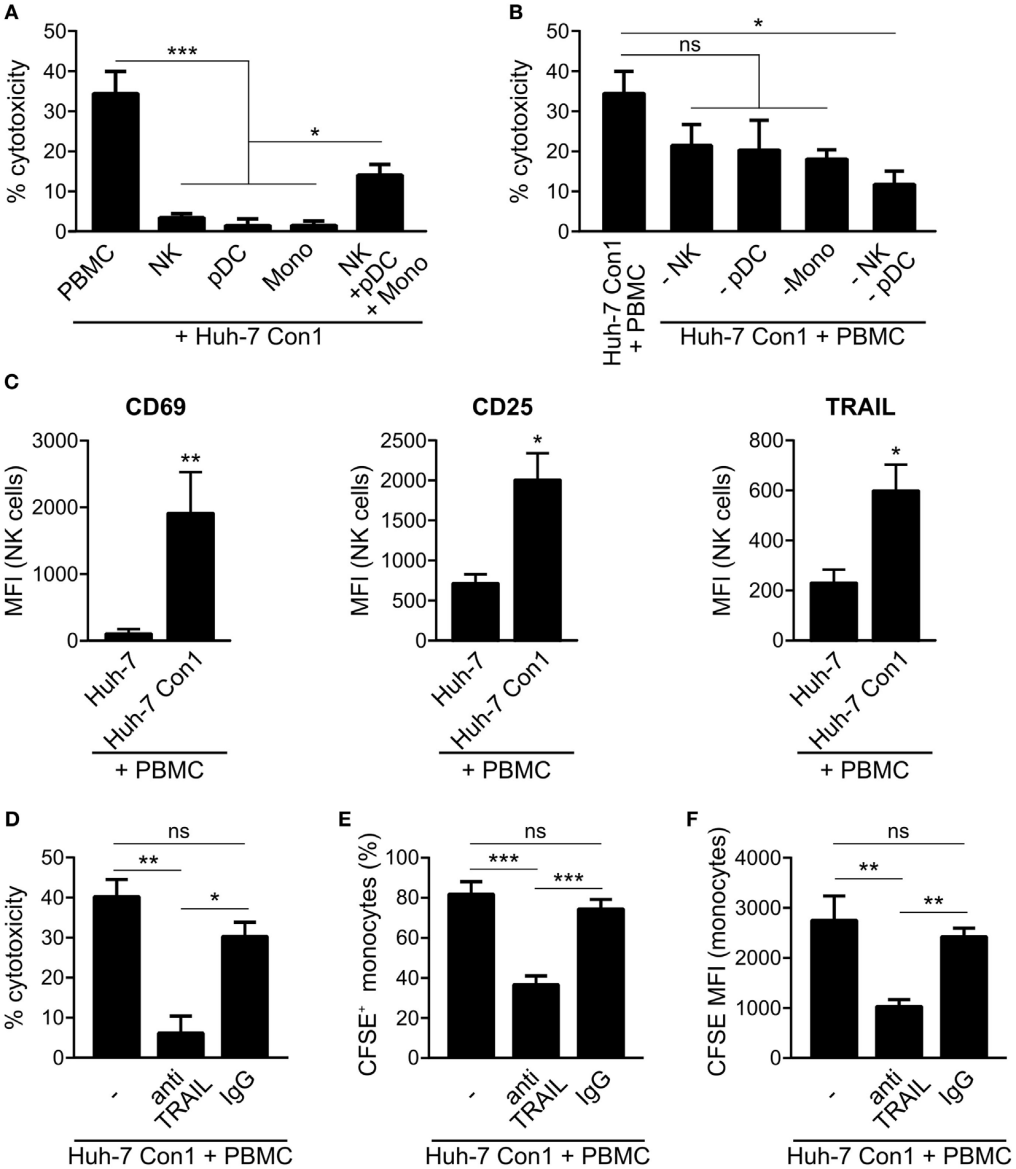
Killing of hepatitis C virus subgenomic replicon cells requires interaction of innate immune cells. **(A)** Huh-7 Con1 cells were co-cultured with peripheral blood mononuclear cells (PBMCs), purified immune cells [natural killer (NK), plasmacytoid dendritic cells (pDC), or Monocytes (Mono)] or with a recombination of purified NK cells, pDCs and monocytes (NK + pDC + Mono) overnight. Supernatants were collected and measured for lactate dehydrogenase (LDH) activity (*n* = 3). **(B)** Huh-7 Con1 cells were co-cultured with PBMCs, PBMCs depleted from NK cells (-NK), from pDCs (-pDC), from monocytes (-Mono), or from NK cells and pDCs (-NK -pDC) overnight. Supernatants were collected and measured for LDH activity (*n* = 4). **(C)** Huh-7 cells were co-cultured with PBMCs overnight. NK cell (CD56^+^, CD3^−^) expression of CD69, CD25, and TRAIL was analyzed by flow cytometry (*n* = 4). **(D–F)** Huh-7 Con1 cells were co-cultured with PBMCs in the presence of an anti-TRAIL antibody or an IgG control antibody (each 10 µg/ml) overnight. LDH release was measured in the supernatant **(D)**, percentage of CFSE^+^ monocytes **(E)**, and carboxyfluorescein succinimidyl ester (CFSE) mean fluorescence intensity (MFI) of monocytes **(F)** was determined (*n* = 4).

As we observed that PBMCs killed HCV SGR cells, we analyzed which cells within PBMCs cause apoptosis in Huh-7 Con1 cells. NK cells, pDCs or monocytes alone were not able to kill Huh-7 Con1 cells, only recombination of purified pDCs, NK cells, and monocytes resulted in killing of Huh-7 Con1 cells (Figure 6A). However, killing was not as efficient as with complete PBMCs, which might be due to functional impairment when purifying single cells. Next, pDCs, NK cells, or monocytes were depleted from PBMCs before setting up the co-culture with Huh-7 Con1 cells. LDH release assays revealed that each cell subset contributed to killing, but none was solely responsible (Figure 6B). Only when both pDCs and NK cells were depleted, killing was significantly reduced. The data confer that interaction of multiple innate immune cells is necessary for an efficient anti-viral response.

### NK Cells Drive Apoptosis in HCV SGR Cells *via* TRAIL Expression

Analyzing the contribution of the different cell subsets in more detail we found an upregulation of the NK cell activation markers CD69 and CD25 in co-culture of Huh-7 Con1 cells with PBMCs. Also, TRAIL expression increased on NK cells in the co-culture setup (Figure 6C). As TRAIL is known to induce apoptosis, we investigated if blocking of TRAIL decreases killing and subsequent uptake of Huh-7 Con1 cells. Indeed, when blocking TRAIL with a specific antibody, killing was reduced from 40 to 6% (Figure 6D). Similarly, the number of CFSE positive monocytes and the CFSE MFI of monocytes were significantly lower (Figures 6E,F).

### Activated PBMCs Only Target HCV SGR Cells, but Not Naïve Huh-7 Cells

**Figure 7:**
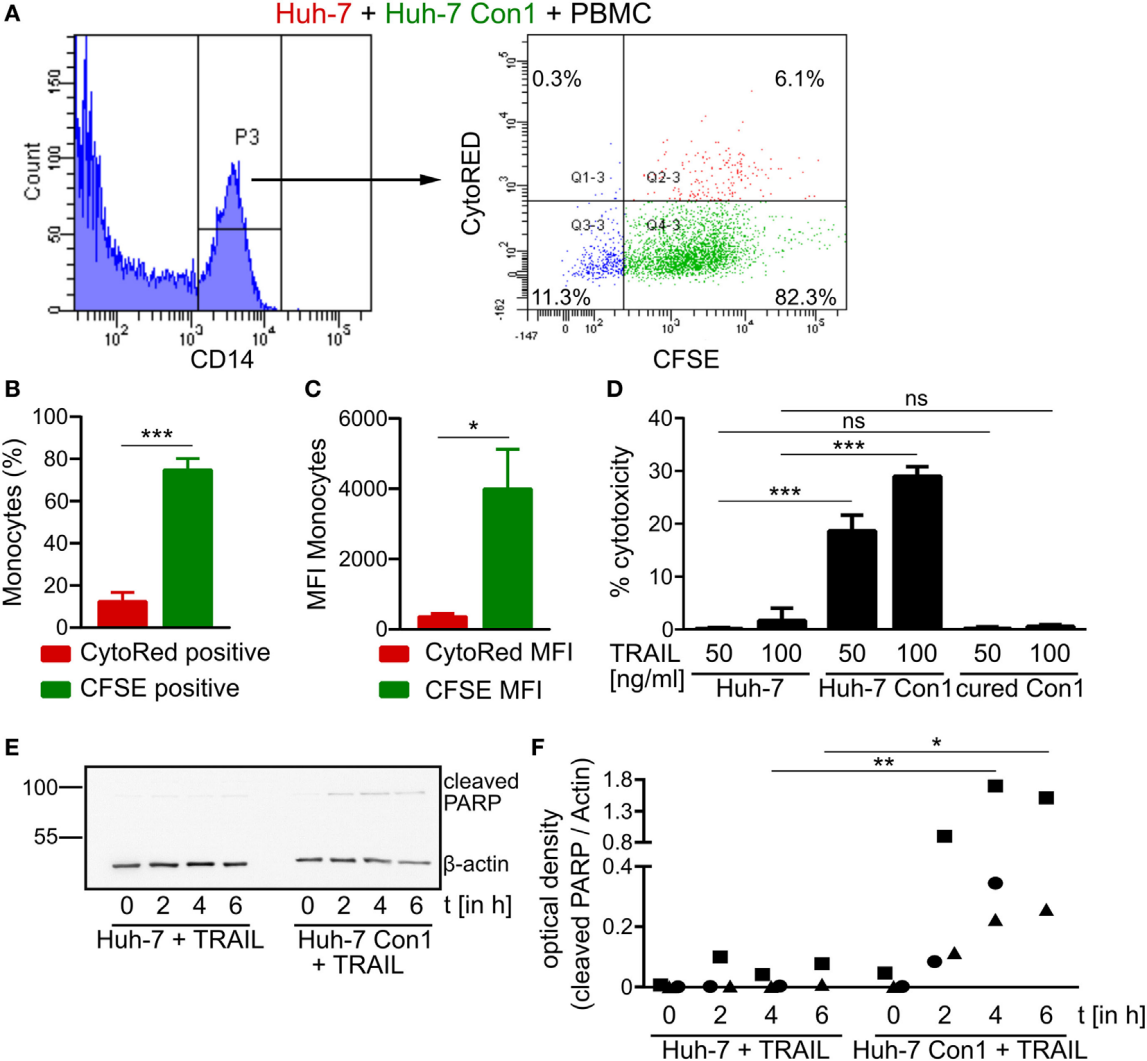
Hepatitis C virus (HCV) specific killing by activated peripheral blood mononuclear cells (PBMCs). **(A–C)** CytoRED stained Huh-7 cells were co-cultured with carboxyfluorescein succinimidyl ester (CFSE) stained Huh-7 Con1 cells and PBMCs overnight. The percentage of CytoRED and CFSE positive monocytes **(B)** as well as the CytoRED and CFSE mean fluorescence intensity (MFI) **(C)** of monocytes were measured (*n* = 3). **(D)** Huh-7 cells were incubated with recombinant TRAIL at the indicated concentrations overnight. Supernatants were measured for lactate dehydrogenase (LDH) activity (*n* = 3). **(E,F)** Huh-7 and cells were incubated with recombinant TRAIL (100 ng/ml) for the indicated time points. Poly (ADP-ribose) polymerase (PARP) cleavage was analyzed by Western Blot (*n* = 2–3).

Since TRAIL expression on NK cells leads to killing of HCV SGR cells, we next determined TRAIL receptor expression in Huh-7 and in Huh-7 Con1 cells. However, no differences in expression were observed for the pro-apoptotic receptors DR4 and DR5 and the anti-apoptotic receptor DcR2 at mRNA levels (Figures S4A–C in Supplementary Material), which was also confirmed by flow cytometry analysis (data not shown). DcR1 was not expressed. As no overt difference in expression of receptors for TRAIL could be observed between Huh-7 and Huh-7 Con1 cells, we wondered whether activated PBMCs would retain their specificity for HCV-infected cells or might then unspecifically kill Huh-7 cells. Therefore, a triple co-culture of Huh-7, Huh-7 Con1 cells and PBMCs was established. In order to verify that the PBMCs were activated in this setup, we analyzed IFNα and IFNγ, which expectedly were induced. Moreover, NK cell activation markers CD25 and CD69 were also upregulated (Figures S5A–D in Supplementary Material). Secretion of IFNs and expression of CD25 and CD69 was lower compared to the co-culture of PBMCs and Huh-7 Con1 cells, as in the triple co-culture only half the amount of Huh-7 Con1 cells was present. Then we analyzed whether Huh-7 cells would be killed in this triple co-culture setup in which PBMCs were activated by the presence of Huh-7 Con1 cells. Huh-7 cells were stained with CytoRed and Huh-7 Con1 cells were stained with CFSE. After overnight co-culture with PBMCs, monocytes were analyzed for CFSE and CytoRed positivity. Strikingly, monocytes turned CFSE positive indicating uptake of Huh-7 Con1 remnants, yet CytoRed staining was only at background levels (Figures 7A–C). Thus, even in a triple co-culture, killing and uptake remained specific for HCV SGR cells. To exclude any effects of the dyes, staining of Huh-7 and Huh-7 Con1 cells was also performed *vice versa* with similar results (Figures S5E–G in Supplementary Material). The results indicate that PBMCs specifically kill HCV SGR cells and despite of their activation by HCV SGR cells they do not target Huh-7 control cells present in the same culture.

### HCV SGR Cells Are Sensitive to TRAIL-Induced Apoptosis

Although Huh-7 and Huh-7 Con1 cells displayed similar TRAIL receptor expression (Figures S4A–C in Supplementary Material), PBMCs specifically killed Huh-7 Con1 cells in a TRAIL dependent manner (Figure 6D). Hence, we hypothesized that HCV SGR cells might be more sensitive to TRAIL induced apoptosis. To test this, recombinant TRAIL was added to Huh-7, Huh-7 Con1 and cured Con1 cells. While Huh-7 and cured Con1 cells were not killed by TRAIL treatment, Huh-7 Con1 cells were killed (Figure 7D). Noteworthy, addition of TNFα neither induced apoptosis in Huh-7 nor in Huh-7 Con1 cells (Figure S6 in Supplementary Material). To confirm the finding of increased TRAIL sensitivity in Huh-7 Con1 cells by an independent assay, cleavage of PARP was examined after addition of recombinant TRAIL. As seen in the PBMC co-culture, cleaved PARP was only detected in Huh-7 Con1 cells (Figures 7E,F). Thus, TRAIL sensitization in HCV SGR cells explains specific killing by PBMCs.

## Discussion

The results of this study highlight the importance of the interplay of different innate immune cells to initiate an efficient, rapid and specific response against HCV-infected cells. The rapid response within the short time frame of an overnight co-culture points out that the anti-viral response is elicited by innate and not by adaptive immunity. Of course adaptive immunity plays an important role for the defense of HCV infection, yet in this study, we focused on the less well explored role of early innate immune responses. Complete PBMCs within 24 h were able to induce apoptosis and clear HCV SGR cells (Figures 2 and 5). In contrast, killing activity of purified subsets of immune cells against HCV SGR cells was substantially lower (Figure 6A). Accordingly, the uptake of Huh-7 Con1 cells by purified monocytes was weaker compared to complete PBMCs. However, activity of purified monocytes against Huh-7 Con1 cells still was higher compared to Huh-7 control cells (Figure 4A), indicating that monocytes play an active role in the anti-viral response. Nevertheless, presence and activation of pDCs and NK cells remained crucial for the most efficient rapid killing *via* TRAIL expression by NK cells (Figure 6), arguing for the need of a precisely controlled interplay.

We observed that an efficient anti-viral response against HCV relied on dual roles of activated immune cells. First, they directly target HCV-infected cells, e.g., by secretion of IFNs (Figures 1A,B) or by upregulation of TRAIL on NK cells (Figure 6). Second, immune cells interact with each other and thereby increase innate activity and specificity. Of note, the observed effects did only occur with HCV SGR but not with DV and HAV SGR cells. The latter two were not killed by PBMCs (Figure 3D). The lack of PBMC activation by DV and HAV SGR cells can be explained by reduced secretion of viral RNA compared to HCV SGR cells.

Natural killer cells cannot be activated directly by HCV SGR cells: it was reported that in the co-culture of HCV SGR cells with PBMCs, NK cells can be activated by IL-18 derived from monocytes (7). Another study confirmed the role of monocytes in NK activation, but observed that pDC derived IFNα was more important to activate NK cells (8). In this study, purified NK cells did not kill Huh-7 Con1 cells (Figure 6A) and did not secrete IFNγ in co-culture (data not shown). But our results show that IFNγ from NK cells increased the uptake of Huh-7 Con1 cells by purified monocytes (Figures 4E,F), proving that NK cells in turn also influence the response of monocytes.

IFNα from pDCs boosted the uptake of HCV SGR cell remnants by purified monocytes even more (Figures 4E,F), indicating that monocyte action is enhanced by pDC activation. Moreover, published data show that TRAIL expression of NK cells is dependent on IFNα (23, 24). It was shown that purified pDCs secrete IFNα in co-culture with HCV-infected cells by recognition of HCV RNA *via* TLR7 (6). We could reproduce activation of purified pDCs in our study (data not shown) and confirmed dependency on endosomal TLRs (Figure 1C). However, pDC activation alone was not sufficient to kill HCV SGR cells, interaction with NK cells and monocytes was additionally necessary (Figure 6A). Thus, IFNα is not sufficient to kill HCV replicating cells, yet contributes by activating additional innate immune players (Figures 4E,F).

**Figure 8:**
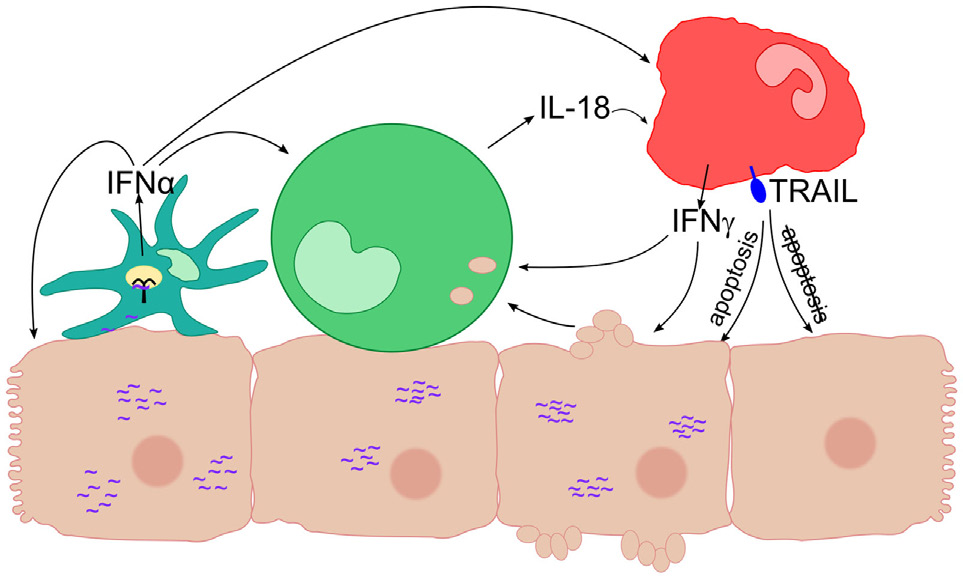
Interaction of different innate immune cells in response to hepatitis C virus subgenomic replicon subgenomic replicon (HCV SGR) cells. Schematic overview of the interactions of innate immune cells leading to anti-viral responses like secretion of interferons (IFNs) and expression of TRAIL. A plasmacytoid dendritic cells (pDC) is depicted in cyan, a monocyte in green, a natural killer (NK) cell in red, viral RNA in purple, and TLR7 inside the pDC in black.

Monocytes can also sense HCV-infected cells and respond by inflammasome activation (7). Furthermore, monocyte-derived macrophages were shown to recognize HCV dsRNA *via* TLR3 (25). In line with this, we observed that purified monocytes were able to take up remnants of Huh-7 Con1 cells (Figure 4A), arguing for a direct recognition of HCV replicating cells by monocytes. The interactions between HCV SGR cells and different innate immune cells are summarized in Figure 8.

Regarding the activation of immune cells by HCV SGR cells, we show that cell–cell contacts are required, since mechanical agitation significantly reduced IFNα secretion (Figure 1E) and no IFNα and IFNγ was produced when PBMCs were separated from HCV SGR cells in a transwell (data not shown). Thus, we confirm previous findings on the importance of cell–cell contacts (6, 21). Dreux et al. showed that also exosomes released from HCV-infected cells can activate purified pDCs; mechanistically, activation of pDCs occured due to transfer of viral RNA (22). However, we did not observe stimulation of complete PBMCs by exosomes (data not shown), which highlights the necessity for cell–cell contacts. We speculate that not only close contacts between immune cells and HCV SGR cells are important but also close interactions between immune cells are necessary for their mutual interaction discussed before. In case of full activation of PBMCs, apoptosis was induced in Huh-7 Con1 cells as shown by cleaved PARP assays and caspase inhibition experiments (Figure 5). Mechanistically we found an upregulation of TRAIL on NK cells in co-culture of PBMCs with Huh-7 Con1 cells (Figure 6C). Although blocking TRAIL significantly reduced killing and uptake of Huh 7 Con1 cells (Figures 6D–F), neither kill nor uptake were completely lost. This suggests that also other mechanisms contribute to killing of HCV SGR cells.

The expression of TRAIL on NK cells in the HCV context has been discussed controversially, since TRAIL on the one hand can kill infected cells but also targets liver explants from diseased patients due to upregulation of TRAIL receptors DR4 and DR5 (26). In contrast, in a different study, no upregulation of DR4 and DR5 was found on hepatocytes *in vivo* during chronic hepatitis (27). Here, we analyzed TRAIL receptor expression in Huh-7 and in Huh-7 Con1 cells and found no difference in pro-apoptotic TRAIL receptors DR4 and DR5 and in the anti-apoptotic DcR2 receptor (Figure S4 in Supplementary Material). Despite similar TRAIL receptor expression, naïve Huh-7 cells were not targeted by PBMCs that were activated by Huh-7 Con1 cells in a triple co-culture (Figures 7A–C), indicating a specific kill of infected cells. This was confirmed by recombinant TRAIL addition to Huh-7 cells, where only Huh-7 Con1 cells were driven into apoptosis (Figures 7D,E). According to a previous study, TRAIL sensitization might arise from mitochondrial damage in HCV-infected cells, inducing a cell intrinsic apoptosis pathway. Together with the extrinsic apoptotic signaling *via* TRAIL, the infected cell is finally driven into apoptosis (28).

Next to the TRAIL sensitization of HCV replicating cells, we speculate that also close interactions between innate immune cells contribute to specific killing of infected cells. We hypothesize that NK cell activation occurs at sites where pDCs and monocytes are activated by sensing HCV-infected cells. Thereby, the interaction of different cells helps to increase specificity by coupling specific innate recognition means (e.g., pDC activation *via* TLR7 or monocyte activation *via* TLR3) to the more unspecific effector means (e.g., TRAIL expression by NK cells).

The necessity of precisely controlled spatial and temporal interactions of multiple innate immune cells might give rise to the speculation that full innate immune activation is difficult to achieve. This could contribute to lack of clearance and chronicity of infection in 70–80% of HCV-infected patients. While in our *in vitro* model, direct interactions between different innate immune cells and with multiple HCV-replicating cells are possible, *in vivo* a longer period of time might be required for activation and interaction of multiple innate immune cells. It could be speculated that in HCV-infected patients that spontaneously clear the virus (around 25%), combined activation of innate immune cells might be established before the virus is able to exert its immune-inhibitory functions described for several immune cells (29).

In summary, this study demonstrates how innate immune cells interact with each other in response to HCV-infected cells. Upon recognition of viral RNA that triggers pDC and monocyte activation, this interaction enables further mutual activation of immune cells. Thereby, an efficient response is initiated that eventually leads to killing of HCV-infected cells *via* TRAIL expression on NK cells and other yet unknown mechanisms. Importantly, we show that innate immune cells activated by HCV do not target uninfected cells. Whether similar interactions between innate immune cells also occur in the infected liver is subject of further analysis. Although not addressed in this study, the combined activation of innate immune cells might also play an important role for the onset and magnitude of adaptive immunity. A rapid and concerted action of innate and adaptive immunity might explain why some patients spontaneously clear HCV before chronic infection is established in which HCV inhibits several immune responses.

## Ethics Statement

This study was carried out in accordance with the recommendations of the local Ethic Committee Heidelberg. All subjects gave written informed consent in accordance with the Declaration of Helsinki. The protocol was approved by the local Ethic Committee Heidelberg.

## Author Contributions

AD and VL developed the concept and design of this study. VK, OG, and TE designed experiments. VK, OG, SE, FL, and GW performed experiments and analysis. VK wrote the article with support from AD and VL.

## Conflict of Interest Statement

The authors declare that the research was conducted in the absence of any commercial or financial relationships that could be construed as a potential conflict of interest.

## Abbreviations

HCV, hepatitis C virus; IFN, interferon; SGR, subgenomic replicon; PBMC, peripheral blood mononuclear cells; pDC, plasmacytoid dendritic cells; NK, natural killer cells; TRAIL, tumor necrosis factor-related apoptosis inducing ligand; LPS, lipopolysaccharide; JFH, Japanese fulminant hepatitis; DV, dengue virus; HAV, hepatitis A virus; G418, geneticin; CFSE, carboxyfluorescein succinimidyl ester; JC1, HCV chimera consisting of the strains J6CF and JFH-1; CD, cluster of differentiation; TNF, tumor necrosis factor; MOI, multiplicity of infection; MFI, mean fluorescence intensity; PARP, poly (ADP-ribose) polymerase; DR, death receptor; DcR, decoy receptor; TLR, toll-like receptor; IL, interleukin; LDH, lactate dehydrogenase.
