# Supplementary material for: Interaction and Mutual Activation of Different Innate Immune Cells Is Necessary to Kill and Clear Hepatitis C Virus-Infected Cells
Source: Front Immunol. 2017 Sep 29;8:1238. doi: 10.3389/fimmu.2017.01238 (PMC5649152; doi:10.3389/fimmu.2017.01238)
Supplement: Supplementary file 1 [file data_sheet_1.pdf]

Supplementary Material

Interaction and mutual activation of different innate immune cells is necessary to kill and clear Hepatitis C virus infected cells

Volker Klöss[1], Oliver Grünvogel[2], Guido Wabnitz[3], Tatjana Eigenbrod[1], Stefanie Ehrhardt[1], Felix Lasitschka[4], Volker Lohmann[2], Alexander Dalpke[1]

[1] Department of Infectious Diseases, Medical Microbiology and Hygiene, University Hospital Heidelberg, Heidelberg, Germany
[2] Department of Infectious Diseases, Molecular Virology, University of Heidelberg, Heidelberg, Germany
[3] Institute of Immunology, Section Molecular Immunology, University of Heidelberg, Heidelberg, Germany
[4] Institute of Pathology, University Hospital Heidelberg, Heidelberg, Germany

Corresponding author: Prof. Dr. Alexander Dalpke, Department of Infectious Diseases, Medical Microbiology and Hygiene, University Hospital Heidelberg, Im Neuenheimer Feld 324, 69120 Heidelberg, Germany
Phone: +49 6221 56 38173, Fax: +49 6221 56 5857 29
E-mail: alexander.dalpke@med.uni-heidelberg.de

Supplementary Figures

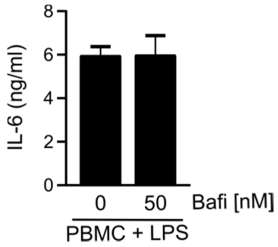

Suppl Figure 1 Bafilomycin is specifically inhibiting endosomal TLRs
PBMCs were stimulated with LPS (0.1 ng/ml) in the presence or absence of Bafilomycin (Bafi). After overnight incubation, cell free supernatants were collected and measured for IL-6 levels by ELISA (n=3).

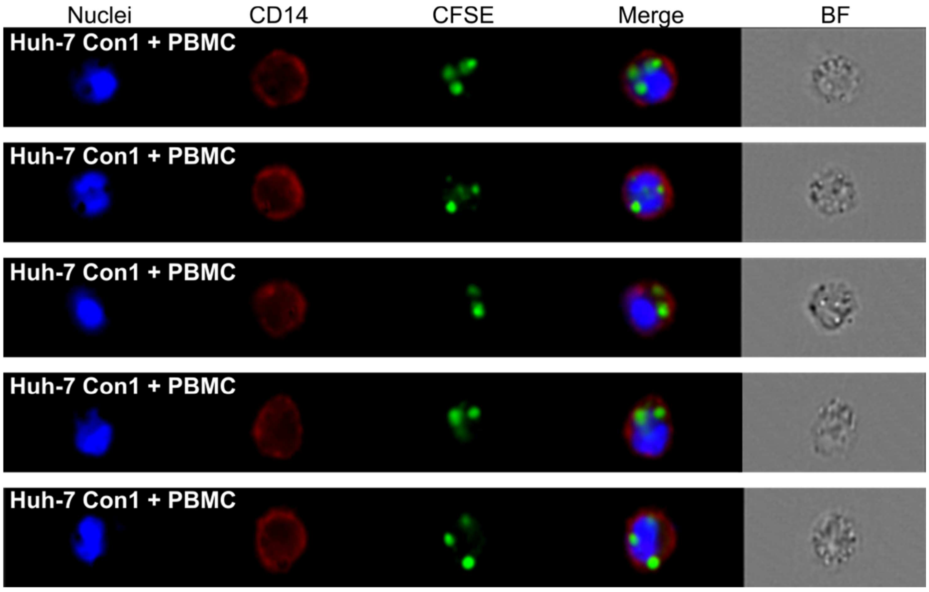

Suppl Figure 2 Phagocytosis of particles from HCV SGR cells by monocytes
Huh-7 Con1 cells were stained with CFSE prior to co-cultivation with PBMCs. PBMCs were added 4 h later and after overnight co-culture cells were harvested for further analysis. Monocytes were stained by CD14 antibody. Uptake of Huh-7 Con1 cells by monocytes is depicted by ImageStream analysis.

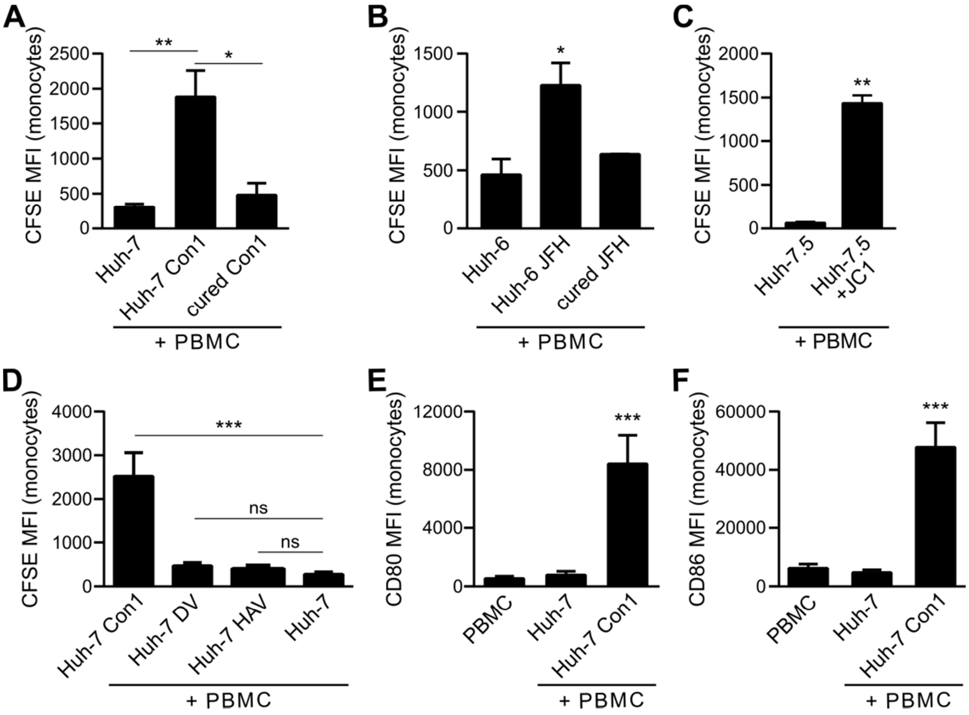

Suppl Figure 3 Phagocytosis by monocytes is specific for HCV

(A-D) PBMCs were co-cultured with CFSE stained cells. After overnight incubation cells were harvested and monocytes were stained with CD14 antibody. To quantify the uptake by monocytes, the CFSE MFI of monocytes was determined by flow cytometry. (A) Co-cultures of PBMCs with Huh-7 cells (naïve, Con1 (HCV genotype 1b) or cured Con1) (n=3). (B) Co-cultures of PBMCs with Huh-6 cells (naïve, JFH (HCV genotype 2a) or cured JFH) (n=3). (C) Co-cultures of PBMCs with Huh-7.5 cells or with HCV infected Huh-7.5 cells (Huh-7.5 + JC1) (n=2). (D) Co-cultures of PBMCs with Huh-7 cells, Huh-7 Con1, Huh-7 DV (Dengue virus) or Huh-7 HAV (Hepatitis A virus) SGR cells. (n=3). (E, F) Huh-7 or Huh-7 Con1 cells were co-cultured with PBMCs overnight. Cells were harvested and monocytes were analyzed for expression of CD80 and CD86 by flow cytometry (n=4).

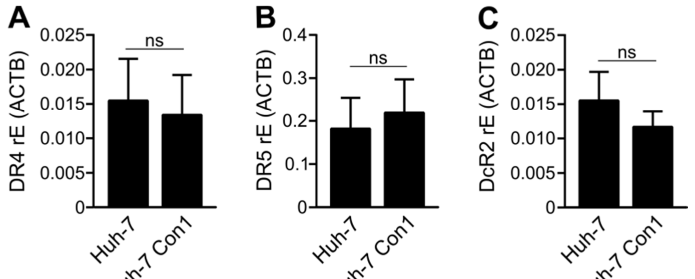

Suppl Figure 4 TRAIL receptor expression on Huh-7 cells

(A-C) Huh-7 and Huh-7 Con1 cells were analyzed for expression of pro-apoptotic TRAIL receptors DR4 (A) and DR5 (B) and for anti-apoptotic TRAIL receptor DcR2 (C) by qPCR.

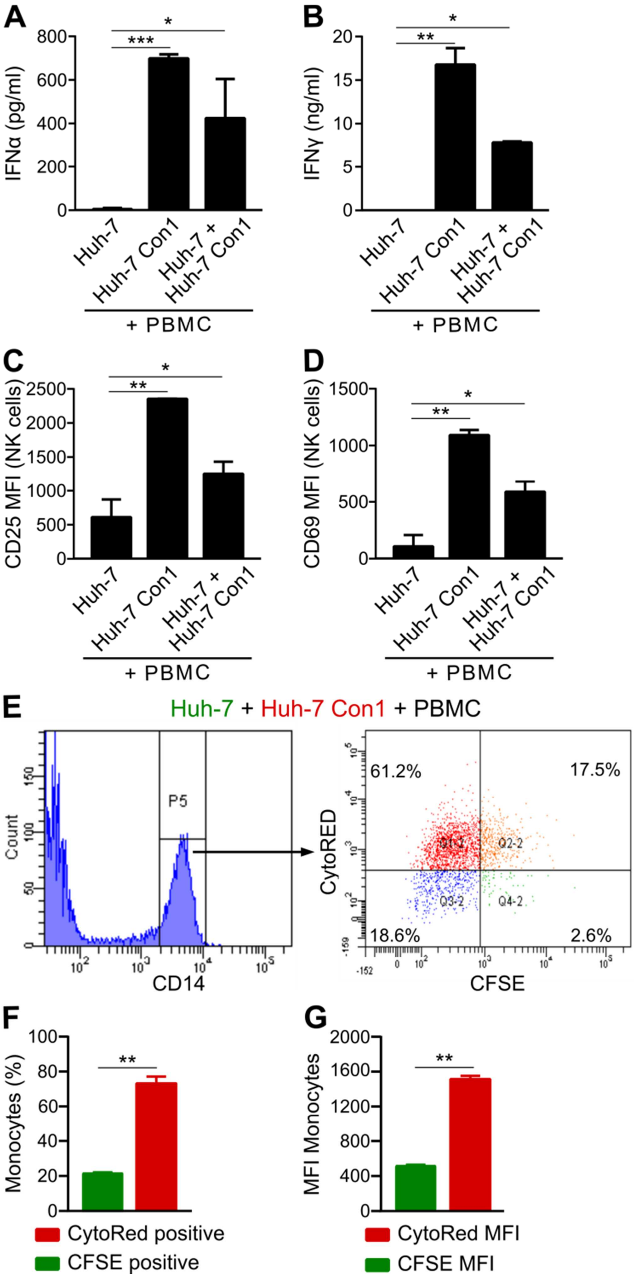

Suppl Figure 5 Innate immune cells are activated in triple co-culture of Huh-7, Huh-7 Con1 and PBMCs

(A-D) PBMCs were co-cultured overnight with Huh-7, Huh-7 Con1 or with Huh-7 and Huh-7 Con1 cells together. Cell free supernatants were harvested and analyzed by ELISA for IFNα (A) and IFNγ (B). Cells were harvested and CD25 (C) and CD69 (D) expression on NK cells was analyzed by flow cytometry (n=2). (E-G) CFSE stained Huh-7 cells were co-cultured with CytoRED stained Huh-7 Con1 cells and PBMCs overnight. Cells were harvested and CD14 positive monocytes were analyzed by flow cytometry for CFSE and CytoRED positivity (E). The percentage of CFSE and CytoRED positive monocytes (F) as well as the CFSE and CytoRED MFI (G) of monocytes were measured (n=2).

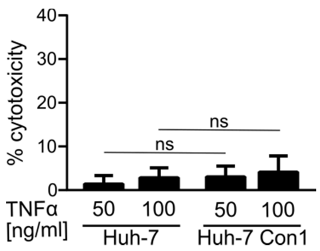

Suppl Figure 6 TNFα does not induce apoptosis of HCV SGR cells

Huh-7 cells (naïve, Con1, cured Con1) were incubated with recombinant TNFα at the indicated concentrations overnight. Cell free supernatants were collected and measured for LDH activity (n=3).

Attachment

Original Image Data

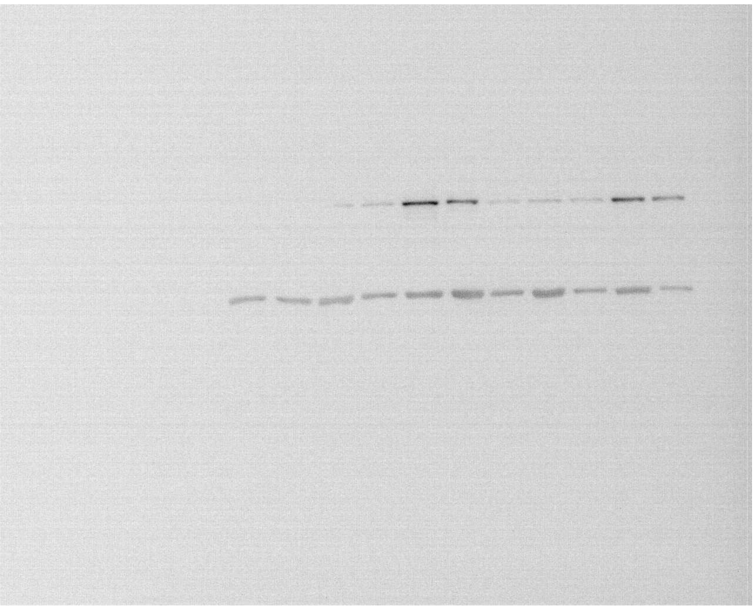

Fig. 5B, Original Blot, lanes 6-10 represent the experimental conditions shown in Fig. 5B (lanes 1-5, lane 11 are different experimental conditions)

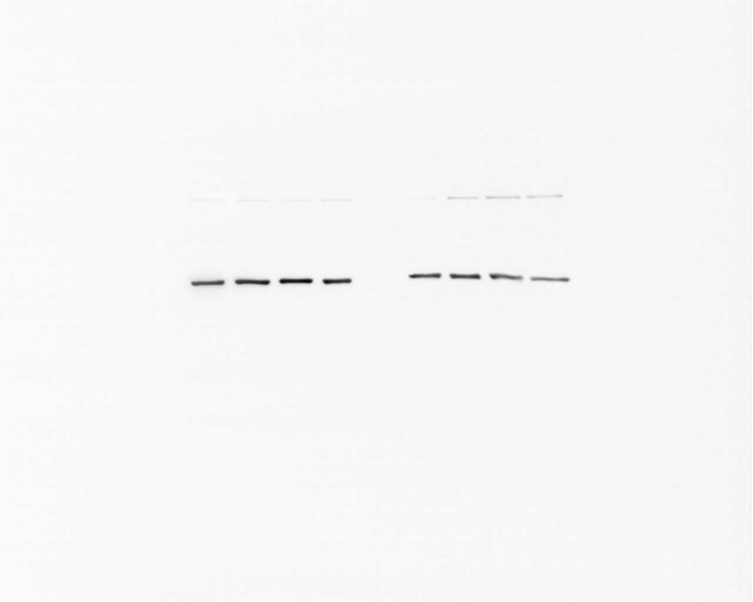

Fig. 7E, Original Blot
